# Notes and News

**Published:** 1893-07-08

**Authors:** 


### Notes and News.

COLLECTED AND COMMUNICATED.

The foundation-stone of the new Halifax Infirmary was laid last week with full Masonic honours, and the town was elaborately decorated. A description and plans of the building will appear in our columns shortly.

The castle and park in Austria, which Baron Nathaniel de Rothschild has presented to Vienna as a consumption hospital, is not to be devoted to this purpose after all, owing to local opposition. Instead it is to be put into a lottery, and the proceeds given to the trustees of the Vienna Hospital.

American experience does not accord with the many disparaging theories as to women's power of gaining offices when competing with men on even ground. Statistics show that in Government departments in the U.S.A. women win their full share of the more responsible and lucrative posts which are equally open to men and women, and it has been noticed that this is especially true with reference to those who have come into the service through the competitive examinations of the Commission. These competitive examinations have been pronounced especially trying to women in the past, but if the robuster physique of the coming generation is able to stand these tests, one of the objections to women occupying posts dependent on such examinations will break down.

Although the fears of the most timid in respect to a visitation of cholera to our shores have been somewhat lulled to rest by the delay of the fell visitant, there is no doubt that a change of weather might prove that our precautions were only too necessary. In France a few cases appear now in this locality and now in that, but Mecca is now the stronghold of the disease. The daily average of deaths is reported at 600, and on one day last week nearly 1,000 were said to have fallen victims. Mecca is not so near a neighbour as Hamburg, but it nevertheless possesses probabilities of spreading the disease through the large number of pilgrims who visit it from many parts of the world. Mecca is thoroughly insanitary, and very little medical aid is forthcoming.

The members of the St. Thomas's and Guy's Hospitals have just presented to their mutual club a very handsome solid silver loving cup. It has three handles, cover, and stands upon three claw feet, and elegantly shaped decoration. On two panels between the handles are depicted in relief faithful views of St. Thomas's and Guy's Hospitals, whilst on the third side are engraved facsimile signatures of the subscribing members. The cup stands on an ebony base, on the sides of which are richly carved replicas of the arms of the two hospitals, and a silver plate bearing the inscription: "St. Thomas' and Guy's Hospitals Club, founded February 14th, 1828. Cup Presented by the Members, 1893." The cup was designed and manufactured by the Goldsmiths and Silversmiths' Company, 112, Regent Street, London, W., in the highest style of their art, and forms a fine specimen of contemporary silverwork.

The Committee of the Homes for Working Boys in London have decided to give a dinner to the 350 residents of the eight Homes on Monday, 10th inst. The Lord Chancellor will take the chair, supported by many influential gentlemen, and an address will be given by Canon Wilberforce. The work of such a society is of so useful a nature that it is difficult to imagine how it would be possible to do without it. The Homes offer an incalculable blessing to boys without friends in London. They are saved the wretchedness and the temptations of lodging houses, and amusements and instruction are provided. The boys pay according to their earnings. A little pamphlet is obtainable at the office of the Homes, 18, Buckingham Street, Strand; it will give those interested in this admirable work full information, and contains details of typical cases of help afforded. We sincerely hope that the interesting festival about to take place will be the means of securing ample funds to carry on and extend these useful Homes.

The Manchester Southern and Maternity Hospital makes especial appeal for aid towards the extension of the building. The hospital, originally founded as a dispensary, gradually grew to its present dimensions, as year by year it became more popular and useful. Eventually a school for midwives and monthly nurses was established, which has become one of the most efficient of its kind in England. Within the last few years the Southern Hospital has become an important auxiliary to the Owens College Medical School. The hospital, now containing 52 beds, proves inadequate to meet the demands upon it, and has also outgrown its site. Moreover, the present fabric possesses many defects. The committee have now the offer of an excellent site, and should the public respond with sufficient generosity a most complete hospital will be erected, a boon to patients and to students alike. Much good medical work has been done by those who owe their instruction to Owens College, and it is to be hoped that those who hold it in grateful memory will help to improve its resources in the future by contributing towards a complete remodelling of the Southern Hospital, and £12,000 will be required for this purpose, of which upwards of £5,000 has already been promised.

